# Mechanical versus manual chest compressions for cardiac arrest: a systematic review and meta-analysis

**DOI:** 10.1186/s13049-016-0202-y

**Published:** 2016-02-01

**Authors:** Hui Li, Dongping Wang, Yi Yu, Xiang Zhao, Xiaoli Jing

**Affiliations:** Department of Emergency, The First Affiliated Hospital of Sun Yat-Sen University, 58 Zhongshan 2nd Road, Guangzhou, Guangdong 510080 China; Department of Organ Transplantation, The First Affiliated Hospital of Sun Yat-Sen University, Guangzhou, 510080 China

**Keywords:** Cardiac arrest, Cardiopulmonary resuscitation, Device, Meta-analysis

## Abstract

**Background:**

The aim of this paper was to conduct a systematic review of the published literatures comparing the use of mechanical chest compression device and manual chest compression during cardiac arrest (CA) with respect to short-term survival outcomes and neurological function.

**Methods:**

Databases including MEDLINE, EMBASE, Web of Science and the ClinicalTrials.gov registry were systematically searched. Further references were gathered from cross-references from articles by handsearch. The inclusion criteria for this review must be human prospective controlled studies of adult CA. Random effects models were used to assess the risk ratios and 95 % confidence intervals for return of spontaneous circulation (ROSC), survival to admission and discharge, and neurological function.

**Results:**

Twelve trials (9 out-of-hospital and 3 in-hospital studies), involving 11,162 participants, were included in the review. The results of this meta-analysis indicated no differences were found in Cerebral Performance Category (CPC) scores, survival to hospital admission and survival to discharge between manual cardiopulmonary resuscitation (CPR) and mechanical CPR for out-of-hospital CA (OHCA) patients. The data on achieving ROSC in both of in-hospital and out-of-hospital setting suggested poor application of the mechanical device (RR 0.71, [95 % CI, 0.53, 0.97] and 0.87 [95 % CI, 0.81, 0.94], respectively). OHCA patients receiving manual resuscitation were more likely to attain ROSC compared with load-distributing bands chest compression device (RR 0.88, [95 % CI, 0.80, 0.96]). The in-hospital studies suggested increased relative harm with mechanical compressions for ratio of survival to hospital discharge (RR 0.54, [95 % CI 0.29, 0.98]). However, the results were not statistically significant between different kinds of mechanical chest compression devices and manual resuscitation in survival to admission, discharge and CPC scores for OHCA patients and survival to discharge for in-hospital CA patients.

**Conclusions:**

The ability to achieve ROSC with mechanical devise was inferior to manual chest compression during resuscitation. The use of mechanical chest compression cannot be recommended as a replacement for manual CPR, but rather a supplemental treatment in an overall strategy for treating CA patients.

## Background

Sudden cardiac arrest (CA) occurs when someone’s heart stops beating unexpectedly. Minimally interrupted, regular and appropriate cardiopulmonary resuscitation (CPR) can keep blood flowing to the victim’s vital organs while the heart is not pumping [1–4]. Unfortunately, even healthcare professionals have difficulty in performing effective CPR persistently, especially in a moving vehicle and in situations of prolonged cardiac arrest [5–8]. Chest-compressions often are too shallow, hands-off time is too long, chest compression rate is less than 90/min, and rescuers fatigue over time [9–12].

Machines have been developed to take over this chest pumping action using pneumatically driven or load-distributing bands (LDBs) mechanisms, because the machines do not pause or get tired, and deliver uninterrupted chest-compressions with a predefined depth and rate [13]. Some studies using those machines for chest compressions have shown that they could achieve intrathoracic pressures higher, improve coronary and systemic perfusion pressures and flows compared with manual CPR in animal models and in a small number of terminally ill patients [14–16]. Some data from human observational studies suggested that mechanical chest compressions might be superior to manual chest compressions in cardiac arrest [16–19]. A few recent meta-analyses could not eliminate all the doubts at this regards because of the paucity of data available and the presence of confounding factors [20–22]. We aimed to investigate which method of chest compression (applying the traditional manual compression vs. using a machine) would result in more lives saved.

## Methods

### Types of studies

A meta-analysis was performed to compare any type of mechanical chest compression device with manual chest compression in the management of patients suffered from CA in out-of-hospital and in-hospital settings. Human prospective randomized controlled studies comparing compressions delivered via any type of powered, automatic mechanical compression device versus manual compression were considered for inclusion. Studies explicitly including patients with CA caused by drowning, hypothermia and toxic substances were excluded.

### Types of outcome measures

The primary outcome for this meta-analysis was return of spontaneous circulation (ROSC) defined as spontaneous palpable pulse. Secondary outcomes included survival to hospital admission for out-of-hospital cardiac arrest (OHCA) patients only, survival to hospital discharge and good neurological outcome after hospital discharge, with Cerebral Performance Category (CPC) scores one or two for both in-hospital and out-of-hospital patients.

### Electronic searches

A search strategy was pursued, using the following search terms: “mechanical”, “manual”, “chest compression” and “cardiopulmonary resuscitation”. Searches were conducted in MEDLINE (1946 to 31 August 2015), EMBASE (1950 to 31 August 2015), Web of Science (including web of science Core Collection, current content connect, BIOSIS Previews, Chinese Science Citation Database and SciELO Citation Index, from the start to 31 August 2015) and the ClinicalTrials.gov registry (on 31 August 2015). We handsearched bibliographies of included papers. The search was inclusive of studies in any language.

### Data collection and analysis

Data selection and data extracted were performed among pairs of independent reviewers, and the results were confirmed by a third review author. Discrepancies were discussed and adjudicated by the team consensus. In reporting the results of this systematic review, the authors have followed the recommended guidelines from the Quality of Reporting of Meta-Analysis (QUOROM) Statement.

### Statistical analyses

Data were checked and entered into the Stata 12.0 (Stata Corp., College Station, TX) database for further analysis. Using a random-effects model, we calculated the risk ratios (RR) and 95 % confidence intervals (CI) for ROSC, arrival to hospital with a spontaneous palpable pulse, survival at discharge and CPC score. The presence of heterogeneity between trials was assessed using the *I*^2^ statistics. Funnel plots and Egger’s regression test were used to assess the potential for reporting bias.

## Results

### Results of the search

**Fig. 1:**
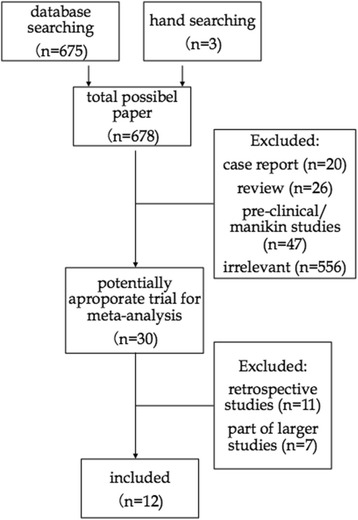
Flow diagram of search criteria and reason for exclusion

In the original review, the comprehensive search identified 678 citations (MEDLINE 118, EMBASE 94, Web of Science 444, clinicaltrials.gov 19, handsearch of references of included papers 3). Two independent review authors reviewed 678 citations by titles; possible citations were selected for review by abstract. After review by abstract, potential relevant were identified and reviewed by full article. After exclusion, 12 studies were found meeting the inclusion criteria, included 9 out-of-hospital studies and 3 in-hospital studies [23–34]. The process was detailed in a PRISMA flow diagram (Fig. 1). Trial characteristics were summarized in the Table 1. Eleven of the articles were published in English and one was Chinese, comprising data from eight countries. Six studies were multiple-center trials, whereas the others were conducted in one single center. Three different mechanisms of mechanical chest compression devices including LDBs (AutoPulse), pistons (LUCAS and Thumper) and pneumatic vests (vest CPR) applied in the 12 studies.

**Table 1:** Summary of studies included in the systematic review

Year	Author	Country		Type of mechanical device	Setting	CPC^a^	ROSC^b^	Survival to admission	Survival to discharge
Out-of-hospital									
1998	Edward T. Dickinson	USA	single center	Thumper	out of hospital	Not reported	1/10:0/7	1/10:0/7	0/10:0/7
2006	Christer Axelsson	Sweden	Multicenter	LUCAS	out of hospital	Not reported	49/105:50/105	35/105:36/105	4/105:2/105
2006	Marcus Eng Hock Ong	USA	single center	AutoPulse	out of hospital	8/101:16/96	101/499:96/278	54/485:58/277	14/486:27/278
2006	Al Hallstrom	USA and Canada	Multicenter	AutoPulse	out of hospital	28/371:12/391	Not report	37/373:23/394	Not reported
2011	David Smekal	Sweden	Multicenter	LUCAS	out of hospital	Not reported	23/73:30/75	15/73:18/75	7/73:6/75
2012	Marcus Eng Hock Ong	Singapore	Multicenter	Autopulse	out of hospital	2/6:13/16	103/459:195/552	6/459:18/552	2/6:13/16
2014	Sten Rubertsson	Sweden, Netherlands and UK	Multicenter	LUCAS	out of hospital	100/1289:108/1300	466/1289:460/1300	809/1289:841/1300	118/1289:117/1300
2015	Sebastian Zeiner	Austria	single center	LUCAS and AutoPulse	out of hospital	113/154:19/31 113/154:2/5	236/655:82/239 236/655:13/44	Not reported	117/655:31/239 117/655:5/44
2015	Gavin D Perkins	UK	Multicenter	LUCAS	out of hospital	168/2815:77/1649	Not reported	193/2819:104/1652	Not reported
In-hospital									
1978	George J. Taylor	USA	single center	Thumper	in hospital	Not reported	10/26:10/24		2/26:3/24
1993	Henry R. Halperin	USA	single center	vest CPR	in hospital	Not reported	3/17:8/17		Not reported
2010	Lu Xiaoguang	China	single center	Thumper	in hospital	Not reported	28/74:42/76		11/74:25/76

^a^ *CPC* cerebral performance category, ^b^ *ROSC* return of spontaneous circulation

### ROSC

**Fig. 2:**
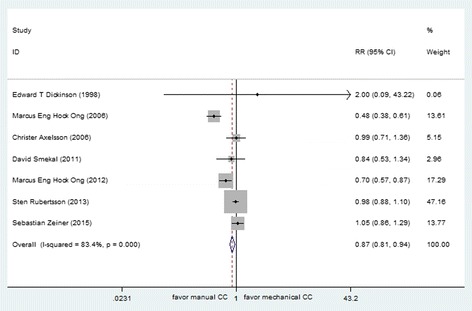
Effect of manual chest compression and mechanical chest compression on ROSC for OHCA patients

**Fig. 3:**
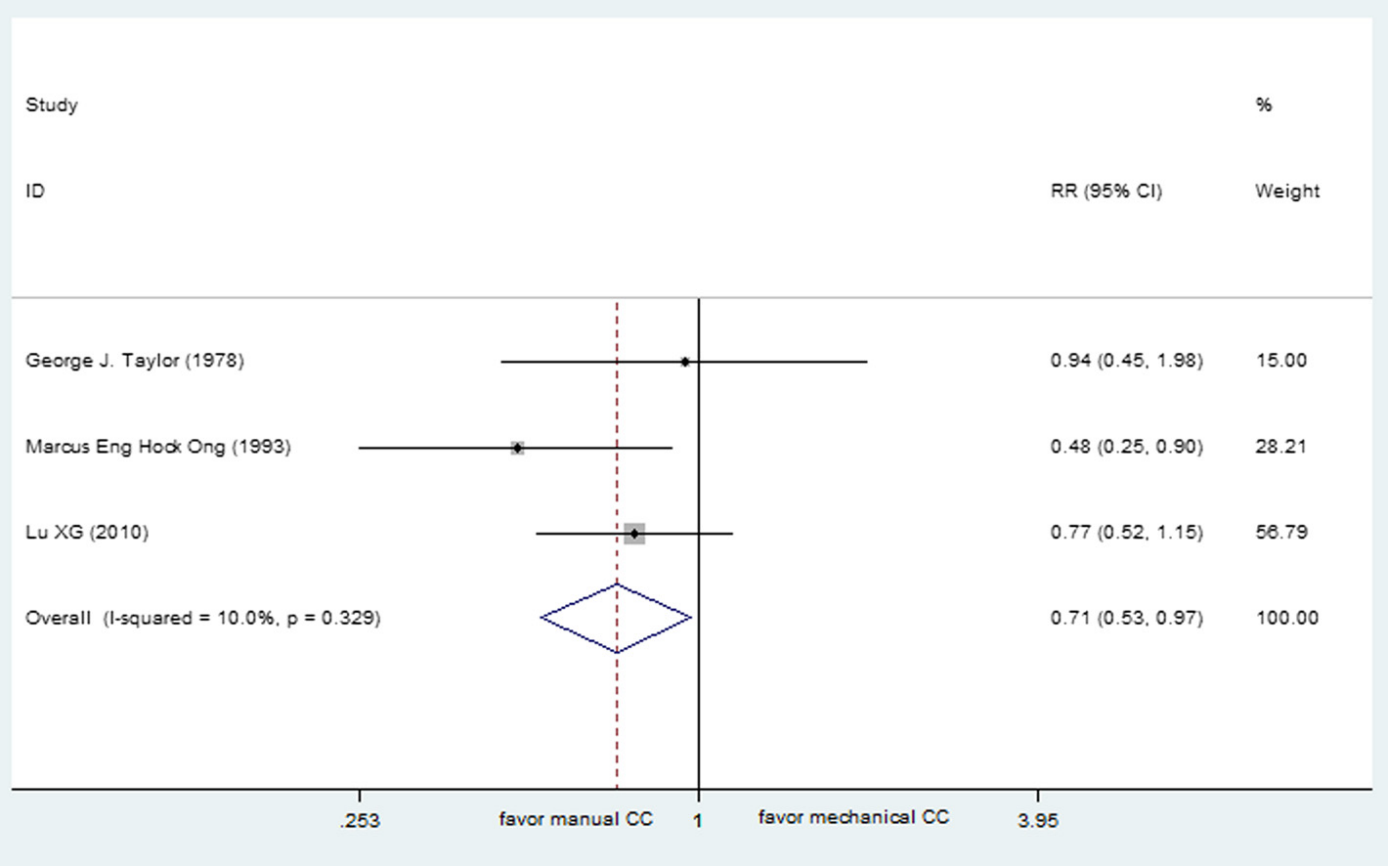
Effect of manual chest compression and mechanical chest compression on ROSC for in-hospital CA patients

ROSC was reported in 10 studies (total *N* = 8886), including 7 out-of-hospital studies (*N* = 8590) and 3 in-hospital studies (*N* = 296). These data were entered into a forest plot respectively, resulting in risk ratio of 0.87 (95 % CI, 0.81, 0.94) for OHCA patients and 0.71 (95 % CI, 0.53, 0.97) for in-hospital CA patients (Figs. 2 and 3). The results suggested harm with mechanical chest compressions for ratio of ROSC in both out-of-hospital and in-hospital setting. The estimated total amount of heterogeneity (*I*^*2*^) was 83.4 % for OHCA patients, which indicated that the treatment effect might not be the same for each device when being compared with manual compression. After analyzed separately according to the device type, a decrease in ratio of ROSC was observed with the use of AutoPulse as compared with manual chest compressions (RR 0.88 [95 % CI, 0.80, 0.96]), while non-significant effect was observed between LUCAS and manual chest compression (RR 1.04 [95 % CI, 0.96, 1.12]) for OHCA.

### Survival to hospital admission

**Fig. 4:**
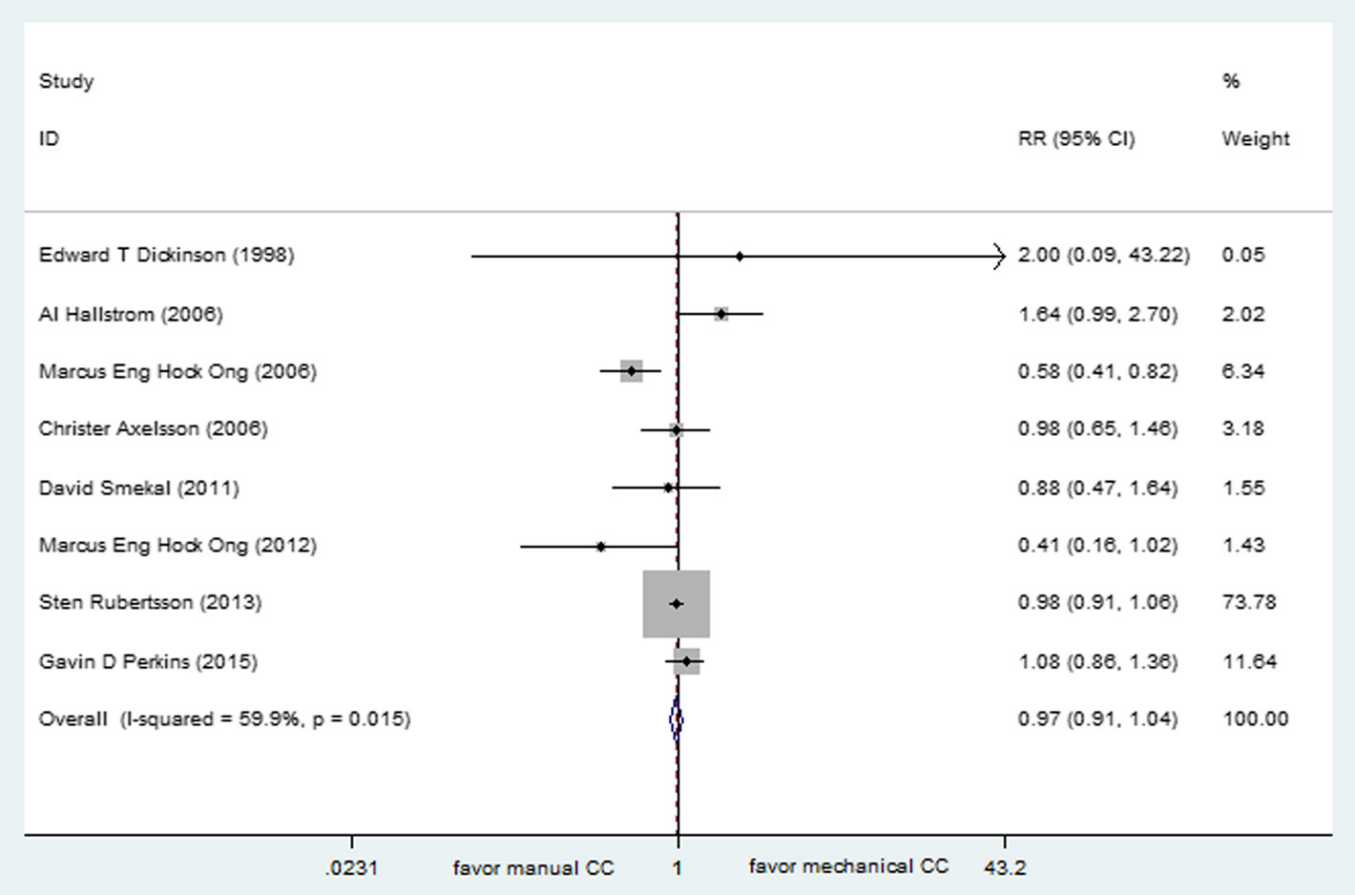
Effect of manual chest compression and mechanical chest compression on survival to hospital admission for OHCA patients

8 studies comprising a total of 9975 OHCA patients met the selection criteria. Although an analysis combining multi-mechanical CPR devices produced an insignificant treatment effect (with an RR 0.97 [95 % CI, 0.91,1.04]) (Fig. 4). The *I*^*2*^ statistic was 59.9 %, the statistical heterogeneity of pooling results were thought to be of relevance to different types of device (AutoPulse, LUCAS and Thumper). Furthermore, when device type was analyzed separately, the treatment effect was not significant for AutoPulse (RR 0.97 [95 % CI, 0.91, 1.02]), for LUCAS (RR 1.02 [95 % CI, 0.94, 1.11]) and for Thumper (0/7:1/10) with manual chest compression, respectively.

### Survival to hospital discharge

**Fig. 5:**
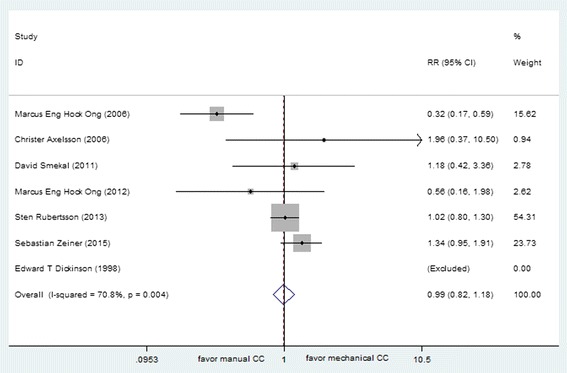
Effect of manual chest compression and mechanical chest compression on survival to hospital discharge for OHCA patients

**Fig. 6:**
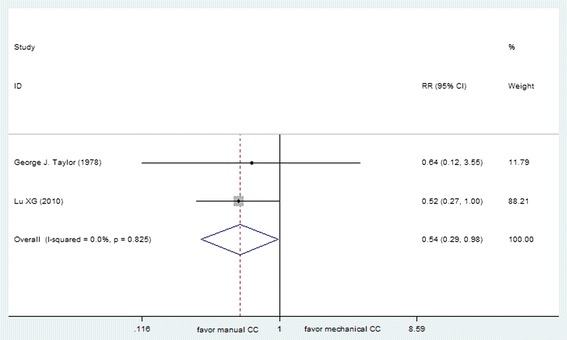
Effect of manual chest compression and mechanical chest compression on survival to hospital discharge for in-hospital CA patients

Several included studies reported data of survival to hospital discharge, including 7 out-of-hospital studies (*N* = 4688) and 2 in-hospital studies (*N* = 200). Data reported in the out-of-hospital studies suggested non-significant effect between manual and mechanical compressions (RR 0.99, [95 % CI 0.82, 1.18]), although the in-hospital studies suggested increased relative harm with mechanical compressions (RR 0.54, [95 % CI 0.29, 0.98]) (Figs. 5 and 6). Considering the heterogeneity due to the type of mechanical device in out-of-hospital studies (*I*^*2*^ = 70.8 %), subgroup analyses were conducted to evaluate the effect of different CPR device on patients with OHCA. The use of any type mechanical chest compression had no significant differences in ratio of survival to discharge with manual chest compression (LUCAS, RR 1.09 [95 % CI, 0.99, 1.19]; AutoPulse, RR 0.96 [95 % CI, 0.89, 1.03]; Thumper, 0/7:0/10).

### CPC

**Fig. 7:**
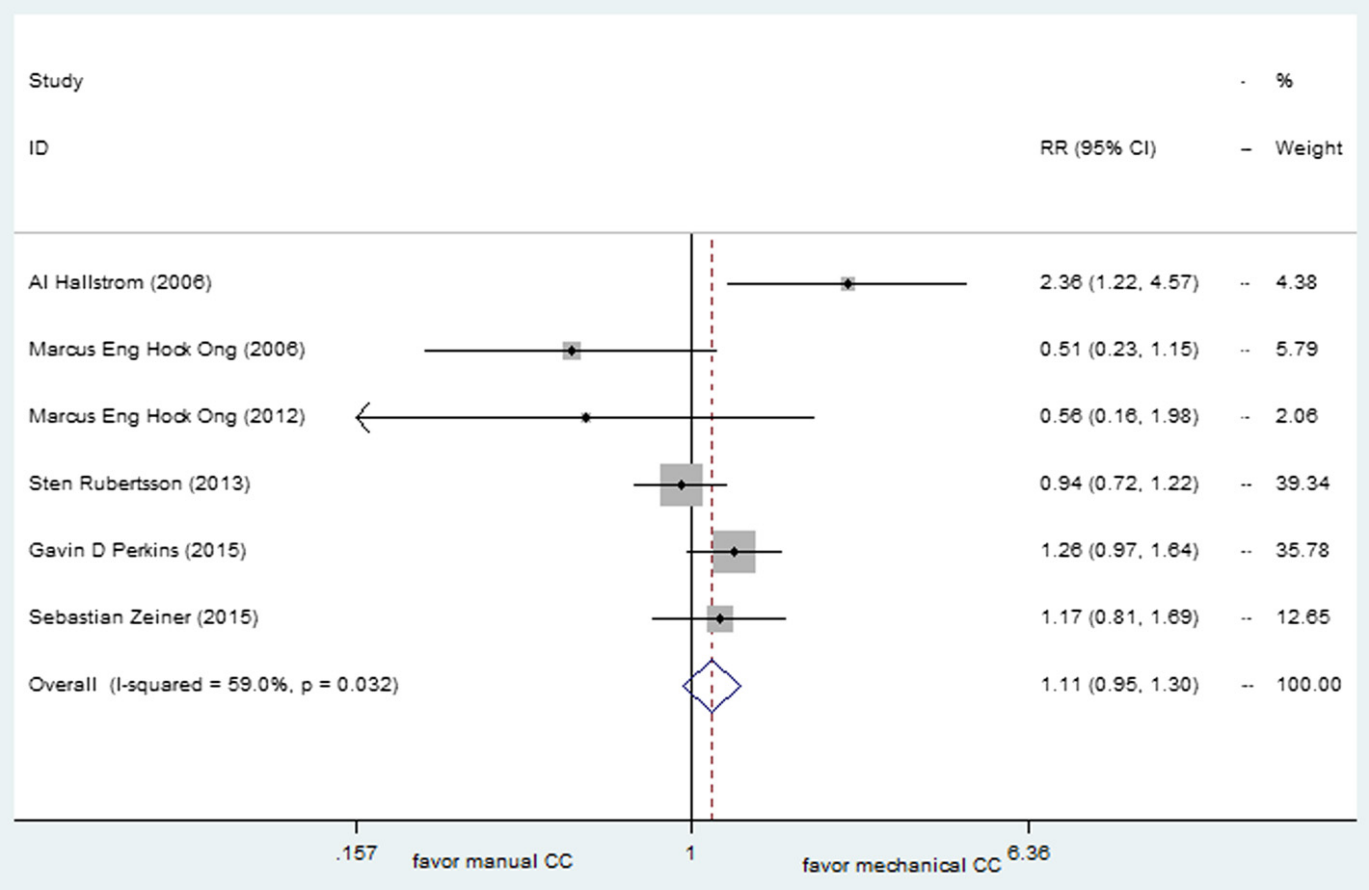
Effect of manual chest compression and mechanical chest compression on CPC for OHCA patients

Six trial including 8825 OHCA patients provided data for the CPC score of this review. The pooled meta-analytic results for good neurological function (defined as a CPC score of one or two) at hospital discharge were not significant (with an RR 1.11 [95 % CI, 0.95, 1.30]), which indicated a similar treatment effect for good neurological outcome with the use of mechanical and manual chest compressions (Fig. 7). The statistical heterogeneity was 59 %. After analyzed separately, the pooling studies reported non-significant increased likelihood of good CPC scores in any type mechanical device comparing with manual compression (LUCAS, RR 1.07 [95 % CI, 0.99, 1.14]; AutoPulse, RR 1.003 [95 % CI, 0.924, 1.090]).

**Fig. 8:**
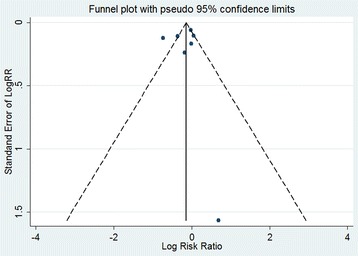
Funnel plot for publication bias for OHCA studies

**Fig. 9:**
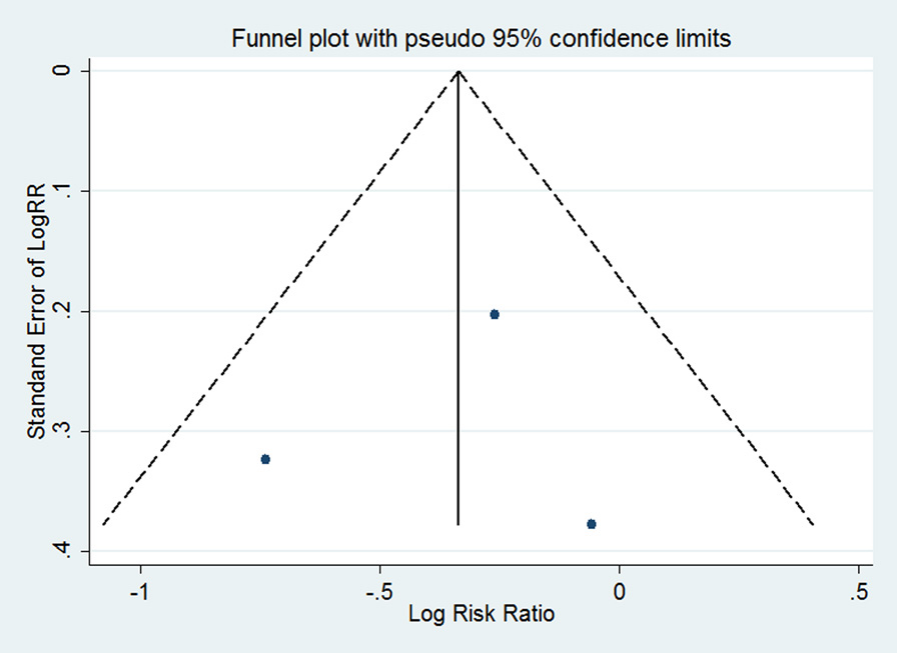
Funnel plot for publication bias for in-hospital CA studies

Meanwhile, the funnel plot indicated the publication bias of the review was acceptable (Figs. 8 and 9). The results of our assessment for risk of bias in included studies were shown in the Table 2. The *P* value from Egger’s regression test showed no significant statistical evidence of publication bias for both out-of-hospital and in hospital CA (*P* = 0.64 for OHCA and *P* = 0.888 for in hospital CA respectively).

**Table 2:** Main quality assessment of included studies

Year	Author	Study design	Description of randomization	Allocation concealment	Description of withdrawals	Blinding outcome assessment
1978	George J. Taylor	RCT	+	–	–	–
1993	Henry R. Halperin	RCT	+	–	–	–
1998	Edward T. Dickinson	RCT	+	–	+	–
2006	Christer Axelsson	Descriptive, non-randomised, controlled trial	–	–	+	–
2006	Al Hallstrom	RCT	+	–	+	–
2006	Marcus Eng Hock Ong	Phased, prospective cohort trial	–	–	+	–
2010	Lu Xiaoguang	RCT	–	–	–	–
2011	David Smekal	RCT	+	–	–	–
2012	Marcus Eng Hock Ong	Phased, prospective cohort trial	–	–	+	–
2014	Sten Rubertsson	RCT	+	–	+	–
2015	Gavin D Perkins	RCT	+	–	+	–
2015	Sebastian Zeiner	Prospective observational study	–	–	–	–

*RCT* randomized controlled trial

## Discussion

The goal of CPR treatment for CA patients is to achieve ROSC and normal neurological function as early as possible while minimizing end-organ damage and dysfunction in the interim. Several animal and human studies have demonstrated an inverse relationship between poor chest compression quality and short-term survival [35–37]. The high quality of CPR has been emphasized in the American Heart Association (AHA) guidelines and emergency cardiovascular care [38]. However, Hightower et al. observed significant fatigue after only one minute of chest compressions on a mannequin. Correct chest compressions were performed 92 % of the time during the first minute, 67.1 % during the second minute and 39.2 % during the third minute [39]. Rescuer fatigue has been identified as an important factor to poor CPR quality. The use of mechanical chest compression devices has been proposed to provide high-quality chest compressions without the interruptions and fatigue, meanwhile the resuscitation efforts can be facilitated by freeing the hands of the rescuer from manual chest compression. For the same reasons, safety increases during transport in a moving ambulance for OHCA patients. Some data from animal and human observational studies suggested that mechanical chest compressions might be superior to manual chest compressions in cardiac arrest [14–19]. The international guidelines published in 2010 suggested the devices could be considered as part of an overall strategy to improve CPR quality [38]. In contrast, several larger RCT studies concluded that mechanical CPR did not result in improved outcomes compared with manual CPR in recent years [32–34]. This systematic review and meta-analysis sought to assess whether there was a difference between mechanical and manual chest compression with respect to CPC, ROSC, survival to hospital admission and discharge.

Twelve studies (8 randomized control trials, 2 phased prospective cohort trials, one phased prospective cohort trial and one descriptive controlled trial) were identified after the search of the literatures, comprising five studies about LUCAS, four about AutoPulse, three about Thumper and one about vest CPR (one paper including both LUCAS and AutoPulse). The publication dates of these included studies span over 40 years. The results of this meta-analysis indicated no difference is found in CPC scores, survival to hospital admission and survival to discharge between manual and mechanical CPR for OHCA patients. The data on achieving ROSC in both of in-hospital and out-of-hospital setting and survival to discharge for in-hospital patients suggested poor application of the mechanical device. Considering the heterogeneity in types of mechanical devices used, we identified studies that reported LUCAS and AutoPulse in ROSC with mechanical chest compressions compared with manual chest compressions respectively, the results shown that OHCA patients receiving manual resuscitation were more likely to attain ROSC compared with AutoPulse; however, the difference was not significant between LUCAS and manual resuscitation. On the whole, available data from clinical trials did not provide sufficient evidence to permit conclusions on the effectiveness of mechanical chest compressions as compared with manual chest compressions, which was similar to the conclusion of another recent review and meta-analysis about OHCA patients [40]; in contrast, for in-hospital CA patients, the use of mechanical chest compression could not be recommended. The limited amount and varied definitions of long-term survival and neurologic function data for each respective study prohibited the use of the data as the outcome variable in this meta-analysis.

Many factors affect the chances of survival after cardiac arrest, including early recognition of arrest, effective cardiopulmonary resuscitation and defibrillation, and post-resuscitation care. A critical issue is the delay between collapse and the start of the intervention. For OHCA patients, witnessed CA receiving bystander basic life support might improve outcome [41]. One of these hypotheses is that interruptions in CPR during device deployment could cause reduced cardiac and cerebral perfusion [1]. It is important to reduce the interval between collapse and the start of chest compression. However, there will always be a delay from the arrival of the rescuer to setting up the equipment and giving the first treatment according to field conditions, which might increase cardiac instability and impair cerebral microcirculation. Secondly, several studies reported that they failed to find any significant difference between manual and mechanical in their outcome of survival or survival to discharge. A possible explanation for these unexpected results advanced by the authors is a Hawthorne effect for manual CPR, which means a type of reactivity in medical rescuers modify or improve an aspect of their behavior in response to their awareness of being observed [42, 43]. Almost all of the included studies did not have the quality of CPR monitoring in place at the time, these data were not collected or reported specifics on how manual CPR was performed in the control group. Although considering the Hawthorne effect, the meta-analysis was unable to show any superiority of mechanical device, which mean the mechanical chest compression might not be better than high-quality manual CPR. Mechanical chest compression should not be seen as a replacement for high quality manual CPR, but rather a supplemental treatment in an overall strategy for treating CA patients. Thirdly, it is also increasingly recognized that although defibrillation is the definitive treatment for ventricular fibrillation, its success is also dependent on adequate circulation [44]. Thus, effective CPR is often a prerequisite for effective defibrillation. Patients presenting in ventricular fibrillation have relatively high survivability with early defibrillation [45]. In practice, the device is usually applied in patients who do not respond to initial defibrillation and require prolonged CPR. Patients in prolonged cardiac arrest who are successfully resuscitated would be expected to have poorer neurological status on discharge compared to those who respond immediately. Finally, management of patients following resuscitation from CA is complex and requires specialized institutions capable of providing advanced care therapies. Patients with ROSC are treated with mild hypothermia to 32 ~ 34 °C for 24 h, if no contraindications are present. Acute coronary angiography is considered during the first 48 h and, if indicated, a percutaneous coronary intervention is performed. Managing these patients achieving ROSC is challenging and requires a structured approach. Clinical recommendations for CPR, including the nature of manual chest compression, have changed drastically over the past 10 to 15 years [38]. The dates of these included studies span over four decades, strategy of post-resuscitation care for ROSC patients has improved. Those might influence the survival of discharge and neurological function over years.

In this meta-analysis, there were some limitations. The first limitation was that some of the included studies were prospective observational trials or phased controlled trials. It was possible that selection bias could be introduced that the paramedics tended to have a lower threshold for initiation of resuscitation. A second limitation was that none of studies were blinded. Because the rescuers who decided when to initiate CPR were not blinded, introducing the possibility of rescuers preferential application of a device to patients thought to have a very poor prognosis in the hope that use of the device might lead to better outcomes than attained with standard care. Thirdly, these included studies were conducted across nearly 50 years, which demonstrated marked heterogeneity in types of mechanical devices used, timing of device application, CPR quality of manual chest compression and post-resuscitation management. Although we had made a subgroup analysis according to the types of mechanical devices (LUCAS, AutoPulse and Thumper) for OHCA patients, the heterogeneity could not be eliminated and would reduce the statistical power of the approach based on the limited data. Finally, very limited data were available from those studies comparing long-term survival and neurological function score, results from these studies were not pooled.

## Conclusions

In this systematic review, the combined meta-analysis of mechanical chest compression devices compared with manual chest compressions shown a better result with manual chest compression in ROSC rates for both out-of-hospital and in-hospital CA patients and survival of discharge for in-hospital CA patients. When analyzed separately, only AutoPulse was found to be inferior to manual chest compressions with ratio of ROSC. We believed that the mechanical chest compression should not be seen as a replacement for manual CPR, but rather a supplemental treatment in an overall strategy for treating CA patients.

## Abbreviations

CA: cardiac arrest
ROSC: return of spontaneous circulation
CPR: cardiopulmonary resuscitation
CPC: Cerebral Performance Category
LDBs: load-distributing bands
RR: risk ratio
CI: confidence interval
